# Supplementary material for: Causal Relationship between Aging and Anorexia Nervosa: A White-Matter-Microstructure-Mediated Mendelian Randomization Analysis
Source: Biomedicines. 2024 Aug 16;12(8):1874. doi: 10.3390/biomedicines12081874 (PMC11351342; doi:10.3390/biomedicines12081874)
Supplement: Supplementary file 1 [file biomedicines-12-01874-s001.zip › Supplementary Figure/Figure S8/Leaveoneout plot of MR analysis for TL to ubm-b-1974.pdf]

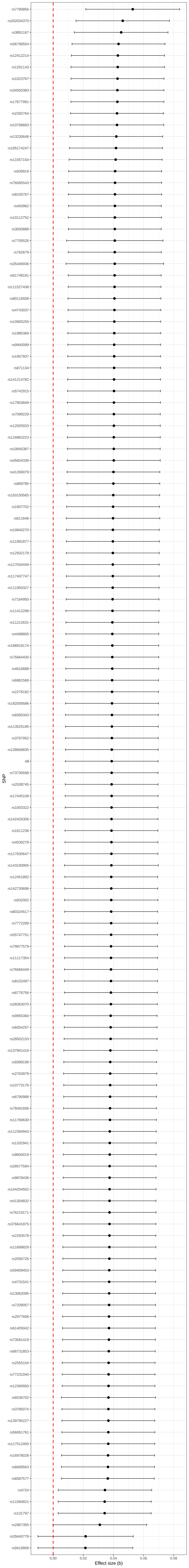

rs7790856
rs202034370
rs3891167
rs56799554
rs12412214
rs1291143
rs1023767
rs34550383
rs17677991
rs2282764
rs10768683
rs13230646
rs185174247
rs11557154
rs939916
rs76065543
rs8105767
rs450962
rs10112752
rs3093888
rs7705526
rs762679
rs35446936
rs61748181
rs111527438
rs80116508
rs4743037
rs10905255
rs1985369
rs9940099
rs1957937
rs871134
rs141214782
rs5742915
rs17803849
rs7099229
rs12925933
rs116863223
rs10845387
rs45604339
rs41269079
rs869785
rs150150565
rs1907702
rs611646
rs10840270
rs11991877
rs12932179
rs117034449
rs117407747
rs111950327
rs7164950
rs11412296
rs11212631
rs4498805
rs188918174
rs75664430
rs4616688
rs6881568
rs2276182
rs182059586
rs6590343
rs113525195
rs3767952
rs139669835
All
rs73730598
rs2538745
rs17445108
rs1003322
rs142426306
rs1611236
rs4530278
rs117630647
rs143190905
rs12451892
rs142730696
rs932002
rs80324517
rs7772289
rs55747751
rs79977579
rs11117354
rs76666449
rs8102497
rs6776756
rs28363070
rs9955360
rs6054257
rs28502153
rs137901416
rs9398196
rs2763979
rs10773176
rs6790988
rs78491606
rs11769630
rs112394943
rs1332941
rs9600019
rs28577594
rs9878436
rs144204502
rs41304832
rs76219171
rs376641875
rs2293579
rs11699829
rs2056726
rs59409453
rs4731541
rs13062095
rs7209057
rs2977608
rs61405042
rs73581419
rs66731853
rs2555104
rs77231040
rs12369950
rs6536702
rs3785074
rs139795227
rs56061761
rs117512405
rs16978028
rs6669563
rs6587577
rs4724
rs11584821
rs131797
rs2967355
rs35640778
rs9419958
SNP
0.00
0.02
0.04
0.06
0.08
Effect size (b)
